# Short-Term Calorie Restriction in Early Life Attenuates the Development of Proteinuria but Not Glucose Intolerance in Type 2 Diabetic OLETF Rats

**DOI:** 10.5402/2011/768637

**Published:** 2011-11-16

**Authors:** Daisuke Nakano, Suwarni Diah, Kento Kitada, Hirofumi Hitomi, Hirohito Mori, Tsutomu Masaki, Hiroyuki Kobori, Akira Nishiyama

**Affiliations:** ^1^Department of Pharmacology, Faculty of Medicine, Kagawa University, Kagawa 761-0793, Japan; ^2^Department of Gastroenterology, Faculty of Medicine, Kagawa University, Kagawa 761-0793, Japan

## Abstract

Childhood obesity is becoming more prevalent; however, the influence of obesity or dieting during childhood on outcomes in adulthood is poorly understood. The aim of this study was to examine the effects of short-term calorie restriction (CR) and high-calorie feeding with high-fat or high-sucrose diets during early life on the development of glucose tolerance and diabetic nephropathy in later life of Otsuka Long-Evans Tokushima fatty (OLETF) rats. Neither high-calorie intake nor CR at 7–13 weeks of age affected glucose tolerance of 27-week-old OLETF rats. On the other hand, proteinuria was lower at 27 weeks of age in CR rats than in the other rats. These results suggest that short-term CR at a young age protects against the development of renal injury in later life. In contrast, short-term high-calorie intake or CR at a young age does not appear to affect glucose metabolism in later life.

## 1. Introduction

A massive increase in the prevalence of defective glucose metabolism, insulin resistance, and cardiovascular morbidity is anticipated in most developed countries [1–3]. Dysfunctional insulin activity is accompanied by complications such as obesity, diabetes, and hypertension, and substantially increases the risk of cardiovascular events. One of the main concerns is the increasing prevalence of obesity in children and adolescents, because it has been reported that the prevalence has reached 50% in several countries. Several studies have shown that body mass index (BMI) during childhood is associated with increased risk of early onset of type 2 diabetes and cardiovascular diseases [4, 5], and that the risk of dying by middle age is 2-3 times higher among obese adolescent girls than among those of normal weight [6]. Although it has been believed that the longer duration of diabetes in obese children is associated with an increased risk of cardiovascular events throughout their lifetime, it is unclear whether childhood obesity itself confers additional risk in later life.

Calorie restriction is one of the possible treatments for diabetes, and it is also well known to prolong the lifespan. Calorie restriction is often used as initial therapy for diabetic patients; however, lifestyle changes are often only started following the diagnosis of diabetes. It is clear that starting calorie restriction during an earlier phase has greater effects on outcomes. However, for obese children, it would be difficult to limit calorie intake throughout their life, and calorie intake often starts to increase after some time. The effects of temporary calorie restriction at a young age on obesity-related problems, such as insulin resistance and diabetes, in later life are still unclear.

Diabetic nephropathy, one of the main complications of diabetes, develops in a time-dependent manner, and the number of patients requiring dialysis or transplantation is steadily increasing. Diabetic nephropathy is considered to be a long-term consequence of diabetes mellitus; therefore, interventions during an early phase are strongly recommended. Both basic and clinical studies have demonstrated that treatment with rennin-angiotensin system (RAS) blockers delays the developments and progression of diabetic nephropathy [7–10]. Interestingly, it has been shown that short-term RAS blockade prevented the development of proteinuria in spontaneous type 2 diabetic rats even if it was conducted just during the prediabetic stage [11]. This supports the belief that early intervention is important to prevent diabetic nephropathy, and also suggests that interventions conducted during early life influence the kidney and protect it from stress in later life. This also suggests that exposure to “diabetic stress” early in life may lead to irreversible biologic changes and adversely affect metabolism in later life.

However, it remains to be determined whether the effects of either excessive or restricted calorie intake during early life are sustained until later life, or whether such interventions affect the incidence of insulin resistance, diabetes, and complications, such as nephropathy. Therefore, the present study was conducted to examine the effect of short-term calorie restriction, high-fat feeding, and high-sucrose feeding during early life on the development of insulin resistance and renal injury in later life in spontaneously type 2 diabetic Otsuka long-Evans Tokushima fatty (OLETF) rats.

## 2. Methods

### 2.1. Animals

All experimental procedures were performed according to the guidelines for the care and use of animals established by the Kagawa University. Six-week-old male OLETF rats (Otsuka Co. Ltd., Tokushima, Japan) were divided into four groups and fed either a standard chow (*n* = 16), standard chow with feeding restriction (70% compared to standard chow-fed group, *n* = 16), high-sucrose (60%) chow (*n* = 16), or high-fat (60%) chow (*n* = 16) between weeks 7 and 13 of age. We used Long-Evans Tokushima Otsuka (LETO) rats as the lean control of OLETF rats. After week 13, the diet was replaced with an unrestricted normal diet and was given until 27 weeks of age. Twenty-four-hour urine samples were collected from rats at 6 (baseline), 13, and 27 weeks of age. All animals underwent a 24-hour acclimatization period in metabolic cages before urine collection. We chose 27 weeks of age because our preliminary experiments showed the OLETF rats developed significant insulin resistance at 25–27 weeks of age. Animals were fasted for 12 hours at 13 and 27 weeks of age and killed to collect retroperitoneal and epididymal adipose tissues and the kidneys. The other tissues were snap frozen in liquid nitrogen and stored at −80°C until use.

### 2.2. Real-Time Reverse Transcription PCR

The mRNA expression of SIRT1 and 3 was analyzed by real-time PCR using an ABI Prism 7000 with Power SYBR Green PCR Master Mix (Applied Biosystems, Foster City, Calif, USA). The oligonucleotide primer sequences for glyceraldehyde-3-phosphate dehydrogenase (GAPDH) and SIRTs were as previously described [12]. All data are shown as the relative differences between control and treated animals after normalization for GAPDH expression.

### 2.3. Oral Glucose Tolerance Test (OGTT)

OGTT was performed 1 week before the end of the experimental period, as previously described [13]. The rats were fasted overnight, and glucose was administered by gavage (2 g/kg). Blood samples were collected from the tail vein before and 10, 30, 60, and 120 min after glucose administration to measure plasma glucose and insulin concentrations.

### 2.4. Other Analytical Procedures

Plasma levels of triglycerides, nonesterified fatty acid (NEFA), total cholesterol, glucose (all: Wako Co., Ltd., Osaka, Japan), and insulin (Rat Insulin ELISA Kit; Shibayagi, Gunma, Japan) were measured using commercially available kits. Urinary protein excretion was determined using a protein assay kit (microTP-test; Wako Co. Ltd.).

### 2.5. Histological Analysis

Kidneys were fixed with 15% formalin and embedded in paraffin. Tissue was sectioned into 20 *μ*m-thick slices and stained with periodic acid Schiff (PAS). Kidney sections were evaluated by light microscopy, as previously described [11].

### 2.6. Statistical Analysis

Values are presented as means ± standard error of the mean. Differences between groups were compared using one-way analysis of variance followed by the Newman-Keuls *post hoc* test. Values of *P* < 0.05 were considered statistically significant.

## 3. Results

**Figure 1:**
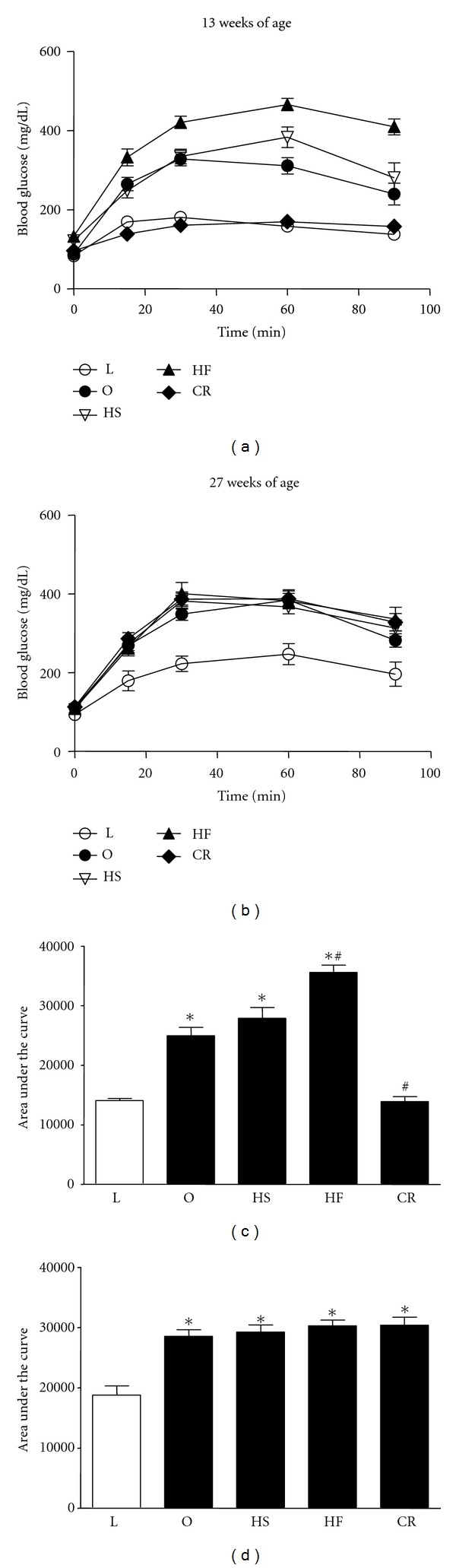
Changes in plasma glucose levels during oral glucose tolerance tests performed at 13 (a) and 27 (b) weeks of age, and the calculated total areas under the glucose curves at 13 (c) and at 27 (d) weeks of age. Compared with the control LETO rats, the normal diet-fed, high-sucrose-fed, and high-fat-fed OLETF rats showed higher glucose levels following the oral glucose load at 13 weeks of age. The changes in blood glucose levels following the oral glucose load in calorie-restricted OLETF rats were almost identical to those in LETO rats at 13 weeks of age. Blood glucose levels were significantly greater in all OLETF rats than in LETO rats, and there were no differences in blood glucose level between any of the OLETF rats at 27 weeks of age. L: LETO + normal diet; O: OLETF + normal diet; HS: OLETF + high-sucrose diet; HF: OLETF + high-fat diet; CR: OLETF + calorie restriction. *P < 0.05 versus LETO rats, ^#^ *P* < 0.05 versus normal diet-fed OLETF rats.

The body weight of normal-diet-fed OLETF rats was significantly greater at 13 and 27 weeks of age than that of LETO rats (Table 1). Short-term high-sucrose feeding did not affect body weight gain in OLETF rats. In contrast, short-term high-fat feeding markedly increased the body weight in OLETF rats at 13 weeks of age, although the difference was abolished at 27 weeks of age. Short-term calorie restriction reduced body weight gain at both 13 and 27 weeks of age. The weight of retroperitoneal and epididymal fat was greater in normal diet-fed, high-sucrose-fed, and high-fat-fed OLETF rats than in LETO rats at both 13 and 27 weeks of age, and retroperitoneal and epididymal fat weight in high-sucrose-fed and high-fat-fed OLETF rats was greater than that in normal-diet-fed rats. The fat weight in the calorie restriction group was similar to that of LETO rats at 13 weeks of age, but it was significantly greater than that in LETO rats at 27 weeks of age. Postprandial, but not fasting blood glucose was greater in the normal-diet-fed OLETF rats than in the LETO rats at 13 and 27 weeks of age. Fasting, but not postprandial, blood glucose concentrations were greater in high-sucrose-fed and high-fat-fed OLETF rats at 13 weeks of age, although this difference was abolished at 27 weeks of age. Postprandial blood glucose in calorie-restricted rats was significantly lower than that in the normal-diet-fed OLETF at 13 weeks of age. There were no differences in postprandial or fasting blood glucose among OLETF rats at 27 weeks of age. The plasma insulin level was increased by high-sucrose and high-fat feeding compared to normal-diet feeding in OLETF rats at 13 weeks of age. No differences in plasma insulin levels were observed among the OLETF groups, including the calorie-restricted group, at 27 weeks of age. Glucose tolerance following oral administration of 2 g/kg glucose in normal diet-fed OLETF rats was impaired compared with that of LETO rats at both 13 and 27 weeks of age (Figure 1). Short-term high-sucrose feeding did not affect glucose tolerance at either 13 or 27 weeks of age. In contrast, short-term high-fat feeding caused marked glucose intolerance at 13 weeks of age, although the difference was abolished at 27 weeks of age. Calorie restriction for 6 weeks markedly improved glucose tolerance at 13 weeks of age, although these improvements were abolished at 27 weeks of age.

**Figure 2:**
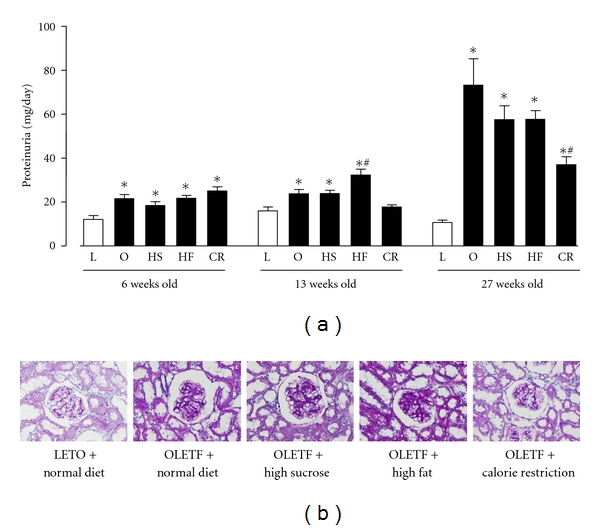
Urinary protein excretion levels (a) and renal morphological changes (b). Proteinuria was greater in all OLETF rats than in LETO rats and was greater in high-fat-fed rats than in normal-diet-fed rats at 13 weeks of age. Proteinuria was further increased at 27 weeks of age in all OLETF rats, although the increment in proteinuria in calorie-restricted OLETF rats was significantly less than that in normal-diet-fed OLETF rats. As shown in (b), the PAS-positive area in the kidney was similar among the 27-week-old rats. L: LETO + normal diet; O: OLETF + normal diet; HS: OLETF + high-sucrose diet; HF: OLETF + high-fat diet; CR: OLETF + calorie restriction. *P < 0.05 versus LETO rats, ^#^ *P* < 0.05 versus normal-diet-fed OLETF rats.

Normal diet-fed OLETF rats developed significant proteinuria at 27 weeks of age (Figure 2(a)). Short-term feeding with a high-fat diet, but not the high-sucrose diet or calorie restriction, caused significant proteinuria in 13-week-old rats. In contrast, neither the high-fat nor the high-sucrose diet affected urinary protein excretion at 27 weeks of age. Interestingly, calorie restriction between weeks 7 and 13 of age reduced proteinuria in 27-week-old rats. The number of glomerulosclerotic lesions detected on PAS-stain tissue sections was markedly increased in normal-diet-fed OLETF rats and in those fed the high-fat or high-sucrose diets, with no differences between these groups (Figure 2(b)). Calorie restriction significantly decreased the number of glomerulosclerotic lesions on PAS-stained tissue sections in OLETF rats.

**Figure 3:**
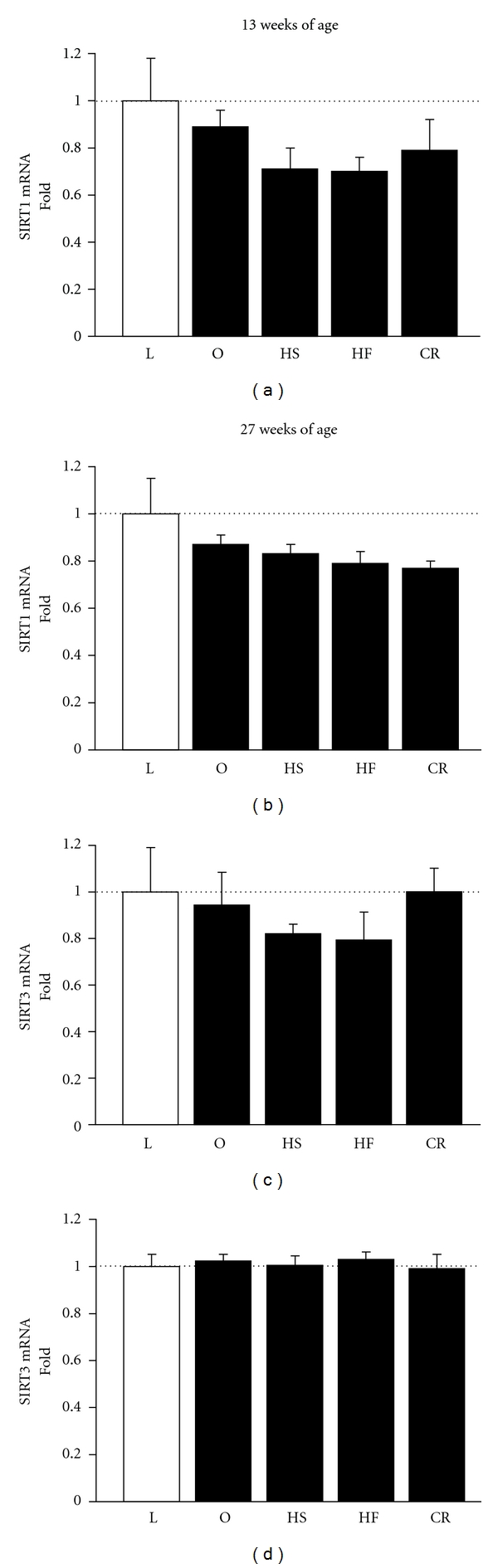
mRNA expression of SIRT1 (a and b) and 3 (c and d) in the kidneys of 13-week-old (a and c) and 27-week-old rats (b and d). There were no differences in either SIRT1 or 3 expression in the kidneys between any of the groups at either time. L: LETO + normal diet; O: OLETF + normal diet; HS: OLETF + high-sucrose diet; HF: OLETF + high-fat dietl CR: OLETF + calorie restriction.

To determine the mechanism by which short-term calorie restriction at a young age delayed or prevented the development of proteinuria in obese animals, we measured sirtuin mRNA expression in the kidney of animals at 13 and 27 weeks of age. However, we found no significant differences in SIRT1 and SIRT3 expression at either 13 or 27 weeks of age between the groups (Figure 3).

## 4. Discussion

The prevalence of obesity in young people is increasing, and many children are now at risk of disorders associated with obesity, particularly endocrine abnormalities such as type 2 diabetes [14]. It appears that obesity in children or adolescents is associated with impaired glucose tolerance and insulin resistance [15, 16], as in adults and that high BMI during childhood increases the risk for cardiovascular events [4]. BMI in childhood and adulthood is strongly correlated; however, an analysis of the association between adolescent BMI and coronary heart disease in adulthood found that the effects were independent of adult BMI [17]. This evidence led us to hypothesize that abnormal calorie intake during young age can lead to irreversible changes in the metabolic systems and insulin resistance in later life. Interestingly, we found that glucose tolerance 14 weeks after stopping the intervention was similar in high-fat-fed, high-sucrose-fed, and calorie-restricted type 2 diabetic rats. It is possible that obesity or leanness during young age might have irreversible effects on total body metabolism because the high-sucrose-fed and high-fat-fed rats were obese with a high body fat ratio at the end of experiment. However, our results showed that the effects of these diets, at least on glucose metabolism, could be diminished by replacing these high-calorie diets with a normal diet. We did not investigate this hypothesis in lean animals, such as LETO rats, because our preliminary experiments showed that short-term high-fat feeding did not significantly worsen glucose metabolism in these rats (data not shown). 

Diabetic nephropathy is a major complication in diabetic patients. It has been shown that the probability of developing albuminuria in patients with childhood-onset type 1 diabetes is similar to that in patients with adult-onset type 1 diabetes [18]. Thus, we expected that the glucose intolerance exhibited in 13-week-old high-fat-fed OLETF rats might worsen the renal lesions in later life. Indeed, the high-fat-fed rats excreted more protein into urine at week 13 of age, although this difference was abolished at 27 weeks of age, as that in glucose tolerance. Surprisingly, the level of proteinuria was reduced by half in calorie-restricted rats, even at 14 weeks after returning to the normal unrestricted diet, indicating that calorie restriction protects against organ injury—the kidney in this study—and that these effects are sustained for longer than those on glucose metabolism. The mechanisms involved in these beneficial effects of short-term calorie restriction are of interest, but we found no possible candidates that are capable of protecting the kidney against type 2 diabetes in later life. We expected the sirtuin family of genes to be a key factor because many studies have shown that increased sirtuin expression contributes to the beneficial effects of calorie restriction on the development of diabetes [19–21]. However, we found no differences in renal SIRT1 or 3 expression among any of the experimental groups at either 13 or 27 weeks of age. We previously reported that RAS components in the kidney were already upregulated at a young age in OLETF rats and that angiotensin AT1 receptor blockade during the prediabetic stage of OLETF rats (4 to 11 weeks of age) prevented the development of diabetic nephropathy in later life without marked effects on body weight, blood glucose, or plasma insulin levels [11]. These findings suggest that inhibition of the renal RAS in the prediabetic stage confers beneficial changes in the kidney similar to that observed following short-term calorie restriction in the present study. The mechanisms underlying these similar effects of calorie restriction and RAS inhibition in the kidney should be investigated in future studies.

## 5. Conclusion

Transient high-fat or high-sucrose feeding in early life does not appear to cause irreversible or sustained changes in either metabolic or kidney function in animals that are genetically obese and type 2 diabetic because of overfeeding. On the other hand, calorie restriction, even if it is temporary, confers protective effects against diabetes-related renal injury. These findings provide further support to the effectiveness of calorie restriction, although it should be noted that it failed to induce sustained benefits on metabolic parameters.

## Figures and Tables

**Table 1:** 

	Time	LETO	OLETF normal diet	OLETF high sucrose	OLETF high fat	OLETF calorie restriction
BW (g)	Baseline	193 ± 3	247 ± 7*	224 ± 4*	216 ± 5*	234 ± 5*
	13 w	393 ± 10	511 ± 14*	524 ± 9*	587 ± 14^∗#^	441 ± 6^∗#^
	27 w	486 ± 17	689 ± 19*	702 ± 15*	705 ± 20*	639 ± 8^∗#^

Fat weight/BW	13 w	0.012 ± 0.001	0.017 ± 0.001*	0.026 ± 0.001^∗#^	0.038 ± 0.001^∗#^	0.011 ± 0.001
	27 w	0.014 ± 0.001	0.022 ± 0.001*	0.027 ± 0.001^∗#^	0.028 ± 0.001^∗#^	0.021 ± 0.001*

Blood glucose (postprandial; mg/dL)	Baseline	98 ± 2	118 ± 5	117 ± 6	123 ± 6	113 ± 6
	13 w	126 ± 2	198 ± 16*	207 ± 17*	218 ± 15*	141 ± 5^#^
	27 w	141 ± 4	265 ± 20*	262 ± 15*	249 ± 24*	229 ± 25*

Blood glucose (fasting; mg/dL)	13 w	84 ± 4	88 ± 4	122 ± 8^∗#^	133 ± 6^∗#^	96 ± 4
	27 w	94 ± 6	112 ± 7	106 ± 6	109 ± 8	113 ± 8

Insulin (fasting; ng/mL)	13 w	0.7 ± 0.2	1.2 ± 0.2	3.4 ± 0.2^∗#^	6.2 ± 0.9^∗#^	1.2 ± 0.3
	27 w	1.9 ± 0.3	5.3 ± 0.4*	5.3 ± 0.5*	6.5 ± 0.6*	5.7 ± 0.5*

*P < 0.05 versus LETO rats, ^#^ *P* < 0.05 versus normal-diet-fed OLETF rats.
